# Left atrial appendage volume is an independent predictor of atrial arrhythmia recurrence following cryoballoon pulmonary vein isolation in persistent atrial fibrillation

**DOI:** 10.3389/fcvm.2023.1190860

**Published:** 2023-06-19

**Authors:** J. Pongratz, L. Riess, S. Hartl, B. Brueck, C. Tesche, U. Ebersberger, T. Helmberger, A. Crispin, M. Wankerl, U. Dorwarth, E. Hoffmann, F. Straube

**Affiliations:** ^1^ Heart Center Munich-Bogenhausen, Department of Cardiology and Internal Intensive Care Medicine, Munich Hospital Bogenhausen, Munich Municipal Hospital Group, Munich, Germany; ^2^ Department of Electrophysiology, Alfried Krupp Hospital, Essen, Germany; ^3^ Department of Medicine, Witten/Herdecke University, Witten, Germany; ^4^ Kardiologie Praxis Erkelenz, Erkelenz, Germany; ^5^ Department of Cardiology, Clinic Augustinum Munich, Munich, Germany; ^6^ KMN—Kardiologie Muenchen Nord, Munich, Germany; ^7^ Department of Radiology, Neuroradiology and Nuclear Medicine, Munich Hospital Bogenhausen, Munich Municipal Hospital Group, Munich, Germany; ^8^ Institute for Medical Information Processing, Biometry and Epidemiology of the Ludwig-Maximilians-University, Campus Grosshadern, Munich, Germany

**Keywords:** catheter ablation, cryoballoon, left atrial appendage, atrial fibillation, left atrium

## Abstract

**Purpose:**

Pulmonary vein isolation (PVI) is the cornerstone of atrial fibrillation (AF) ablation in persistent AF (persAF), and cryoballoon PVI emerged as an initial ablation strategy. Symptomatic atrial arrhythmia recurrence following successful PVI in persAF is observed more frequently than in paroxysmal AF. Predictors for arrhythmia recurrence following cryoballoon PVI for persAF are not well described, and the role of left atrial appendage (LAA) anatomy is uncertain.

**Methods:**

Patients with symptomatic persAF and pre-procedural cardiac computed tomography angiography (CCTA) images undergoing initial second-generation cryoballoon (CBG2) were enrolled. Left atrial (LA), pulmonary vein (PV) and LAA anatomical data were assessed. Clinical outcome and predictors for atrial arrhythmia recurrence were evaluated by univariate and multivariate regression analysis.

**Results:**

From May 2012 to September 2016, 488 consecutive persAF patients underwent CBG2-PVI. CCTA with sufficient quality for measurements was available in 196 (60.4%) patients. Mean age was 65.7 ± 9.5 years. Freedom from arrhythmia was 58.2% after a median follow-up of 19 (13; 29) months. No major complications occurred. Independent predictors for arrhythmia recurrence were LAA volume (HR 1.082; 95% CI, 1.032 to 1.134; *p* = 0.001) and mitral regurgitation ≥ grade 2 (HR, 2.49; 95% CI 1.207 to 5.126; *p* = 0.013). LA volumes ≥110.35 ml [sensitivity: 0.81, specificity: 0.40, area under the curve (AUC) = 0.62] and LAA volumes ≥9.75 ml (sensitivity: 0.56, specificity 0.70, AUC = 0.64) were associated with recurrence. LAA-morphology, classified as chicken-wing (21.9%), windsock (52.6%), cactus (10.2%) and cauliflower (15.3%), did not predict outcome (log-rank, *p* = 0.832).

**Conclusion:**

LAA volume and mitral regurgitation were independent predictors for arrhythmia recurrence following cryoballoon ablation in persAF. LA volume was less predictive and correlated with LAA volume. LAA morphology did not predict the clinical outcome. To improve outcomes in persAF ablation, further studies should focus on treatment strategies for persAF patients with large LAA and mitral regurgitation.

## Introduction

Atrial fibrillation (AF) is the most common sustained arrhythmia with clinical significance worldwide and is strongly associated with an increased stroke rate, heart failure, and overall mortality (1). Ectopic triggers originating from the pulmonary veins (PV) are not only the main cause of AF in paroxysmal AF (PAF), but also in persistent AF (persAF) patients (2). Therefore, the cornerstone of catheter ablation is the electrical isolation of pulmonary veins (PVI). Current guidelines recommend two different techniques: a) the point-by-point radiofrequency ablation (RFA) or b) the single-shot cryoballoon ablation (CBA) approach (3). Although most patients benefit from a PVI only strategy, 31% of patients suffer from atrial arrhythmia recurrence after a mean follow-up (FU) of 16.7 ± 3.0 months (4). The main pathophysiological mechanism of atrial arrhythmia recurrence after the ablation procedure is the reconnection of previously isolated PV (5). Nevertheless, in patients with late atrial arrhythmia recurrence, 20% showed also non-PV triggers in redo procedures (6). In some patients, fibrotic atrial cardiomyopathy and left atrial substrate allows AF to sustain and complex atrial flutter is frequently observed (7). The randomized STAR-AF II study and the randomized DECAAF II trial have demonstrated that neither additional empirical lines or complex fractionated atrial electrograms nor late gadolinium enhanced magnetic resonance imaging (MRI) based substrate ablation in addition to PVI improves outcomes (8, 9). There is a need to characterize those patients prior to ablation who would benefit from additional ablation (8, 10).

During the last decade multiple predictors for atrial arrhythmia recurrence following PVI have been identified: age, female sex, left atrial volume, AF history, left ventricular systolic function, structural and valvular heart disease or diabetes type 2 (11–13). Recently, the left atrial appendage (LAA) has been identified as an extra-PV source of AF (14, 15), and LAA electrical isolation has been proposed as a potential target in AF ablation (14). However, it is unclear if LAA size and morphology could predict recurrences following PVI in persAF. This subgroup of AF is of special interest as atrial arrhythmia recurrence is higher and thus, ablation strategies for PVI non responders need to be defined more precisely (16).

The present study sought to identify predictors of atrial arrhythmia recurrence after cryoballoon PVI in patients suffering from persAF including a variety of LA and LAA features and measurements derived via pre-procedural imaging as well as clinical characteristics.

## Methods

### Study design and objectives

This is a sub-analysis of persAF patients of the previously published prospective observational single-center study with a blinded retrospective analysis of cardiac computed tomography scans by Straube et al. (17). PersAF was characterized according to current AF guidelines with at least one single episode >7 days of continuous AF, including episodes terminated by cardioversion (drugs or electrical cardioversion) after 7 days (3). Patients with PAF were excluded. Atrial arrhythmia recurrence was defined as the re-emergence of PAF, persAF or atrial tachycardia following a three-month blanking period post ablation. Instances of typical right atrial flutter were not considered atrial arrhythmia recurrence. Additionally, patients without atrial arrhythmia recurrence were required to be free of antiarrhythmic medication.

The primary objective was the evaluation of atrial arrhythmia recurrence after initial CBA in patients suffering from persAF and the analysis of associated clinical or anatomical independent risk factors. The secondary endpoint included the safety of the procedure. For analysis the overall study population was divided into two groups: Group A comprised patients with atrial arrhythmia recurrence; Group B patients without atrial arrhythmia recurrence. Subsequently, baseline characteristics, procedural data and measurement parameters were compared. Multivariate analysis was then used to determine independent risk factors for atrial arrhythmia recurrence.

### Pre-procedural diagnostics

Before the ablation procedure intracardiac thrombus formation was excluded by transesophageal echocardiography (TEE). Furthermore, a pre-procedural 64-slice computed tomography scanner (Brilliance 64, Philips Medical Systems, Cleveland, Ohio, USA) that incorporated retrospective ECG gating and 3D reconstruction was utilized to identify individual LA anatomy. Indication for CCTA was based on the clinical presentation of the patient to exclude coronary artery disease, and was performed if there was no recent coronary angiography performed prior to ablation in patients with low to medium risk. Scanning was achieved at 120 kVp and an effective tube current of 600 mAs. The slice collimation was 64 × 0.625 mm, with a gantry rotation time of 0.4 s and a pitch of 0.2. Images were reconstructed at increments of 0.45 mm with a 0.9 mm slice thickness. For contrast enhancement 80 ml of contrast agent (Imeron 400 MCT, Iomeprol 81.65 g/100 ml, Bracco, Konstanz, Germany) was injected at a flow rate of 5 ml/s and followed by a 50-ml saline flush.

### Cryoballoon ablation

A comprehensive description of the procedure has been provided in prior studies (17–19). PVI was accomplished utilizing the 28-mm CB as the preferred balloon size (CBG2: Arctic Front Advance, Medtronic Inc., Minneapolis, MN, USA), which was introduced into the LA over a 15F steerable sheath (Flexcath Advance™, Medtronic Inc., Minneapolis, MN, USA). In case PVI was not achieved, the 23-mm CB size was permitted solely for small PV ≤21 mm. When deemed necessary to complete PVI, radiofrequency (RF) or cryotip touch-up ablations were applied. A 3D reconstruction of the LA via CT was available peri-procedurally to identify PV variants and to guide the operator. Intracardiac echocardiography (ICE; Vivid I, GE Healthcare EUROPE, GE Ultraschall Deutschland GmbH, Solingen, Germany) was used to safely guide transseptal puncture. ICE was additionally used to guarantee ideal balloon placement and vessel occlusion next to PV angiography, and to visualize diaphragmatic motion during right-sided PV ablation if palpation of the diaphragm was difficult. For CB positioning and the evaluation of real-time PV potentials, an 8-pole micro-circular mapping catheter (20-mm Achieve™ or Achieve Advance™ Mapping Catheter, Medtronic Inc.) was used. The refrigerant supply was initiated after the confirmation of optimal vessel occlusion. PVI was performed with at least one freeze-thaw-freeze cycle per vein. If PV potentials could be recognized during the ablation procedure, the time to PV isolation (TTI) was determined. In veins with TTI ≥45 s, or, if no TTI could be detected, an extra freeze was applied. The standard application time was 180 or 240 s, respectively. To confirm PVI, PV potentials were mapped before and after each freeze cycle and again 15 min after the last freeze. As a precaution, the temperature limit for right sided PV was −55°C. In all patients an endoluminal esophageal temperature probe (SensiTherm™, St. Jude Medical, Saint Paul, MN, USA) was used with a cut-off of ≤15°C. To decrease the risk of phrenic nerve (PN) palsy during ablation of the right PVs PN pacing (1,200 ms cycle length) and manual diaphragm examination were conducted.

### Periprocedural management

To ensure an activated clotting time between 300 and 400 s throughout the entire procedure, heparin was administered intravenously with measurements taken at least every 30 min while in the LA. Protamine was given to decrease the risk of bleeding prior to sheath removal. After one-hour, oral anticoagulation was resumed or continuous unfractionated heparin was started with a target partial thromboplastin time of 60–80 s until the next morning. Finally, on the day after the procedure, oral anticoagulation was restarted and continued according to the CHA_2_DS_2_-VASc-Score.

### Post-procedural management and follow-up

Post intervention, all patients received transthoracic echocardiography (TTE) to exclude pericardial effusion as well as extended monitoring with Holter studies for at least 24–48 h. In case of symptoms, additional electrocardiographic and Holter studies were proceeded up to 7 days. Further follow-up studies were arranged to screen for symptomatic or asymptomatic atrial arrhythmias after 1, 3, 6, 9 and at least 12 months. In collaboration with the referring physicians, detailed questionnaires (15 questions) via mail and telephone calls were conducted for each case to ensure best possible follow-up. If recurrence was suspected the referring physician was contacted immediately to validate the diagnosis. Failures were only classified as recurrences outside of the 90-day blanking period.

### Statistical analysis

All acquired data was entered into Microsoft Excel 2016 (Microsoft Corp., Redmond, WA, USA) and subsequently transferred into SPSS version 25 (IBM Corp., Armonk, NY, USA) and R [R Core Team (2022). R Foundation for Statistical Computing, Vienna, Austria] for statistical analysis. Categorical parameters were illustrated as numbers and percentages. Continuous variables were expressed as means with standard deviations (SD) or as medians with quartiles in accordance with the Shapiro-Wilk test. Univariate Cox Regression as well as Kaplan Meier plots with log-rank tests were used to identify relationships between measurement parameters and atrial arrhythmia recurrence after CBA. To evaluate general correlations between all variables and to avoid problems regarding multicollinearity in multivariate models, Pearson's and Spearman's correlation coefficients were calculated. A stepwise multivariate Cox regression model with bidirectional elimination was used to identify the main independent risk factors, and only parameters with highest univariate significance were included. Cut-off values were calculated by a receiver operating characteristic analysis (ROC). Statistical significance was defined as *p* ≤ 0.05.

## Results

### Data acquisition, management, and quality control

**Figure 1:**
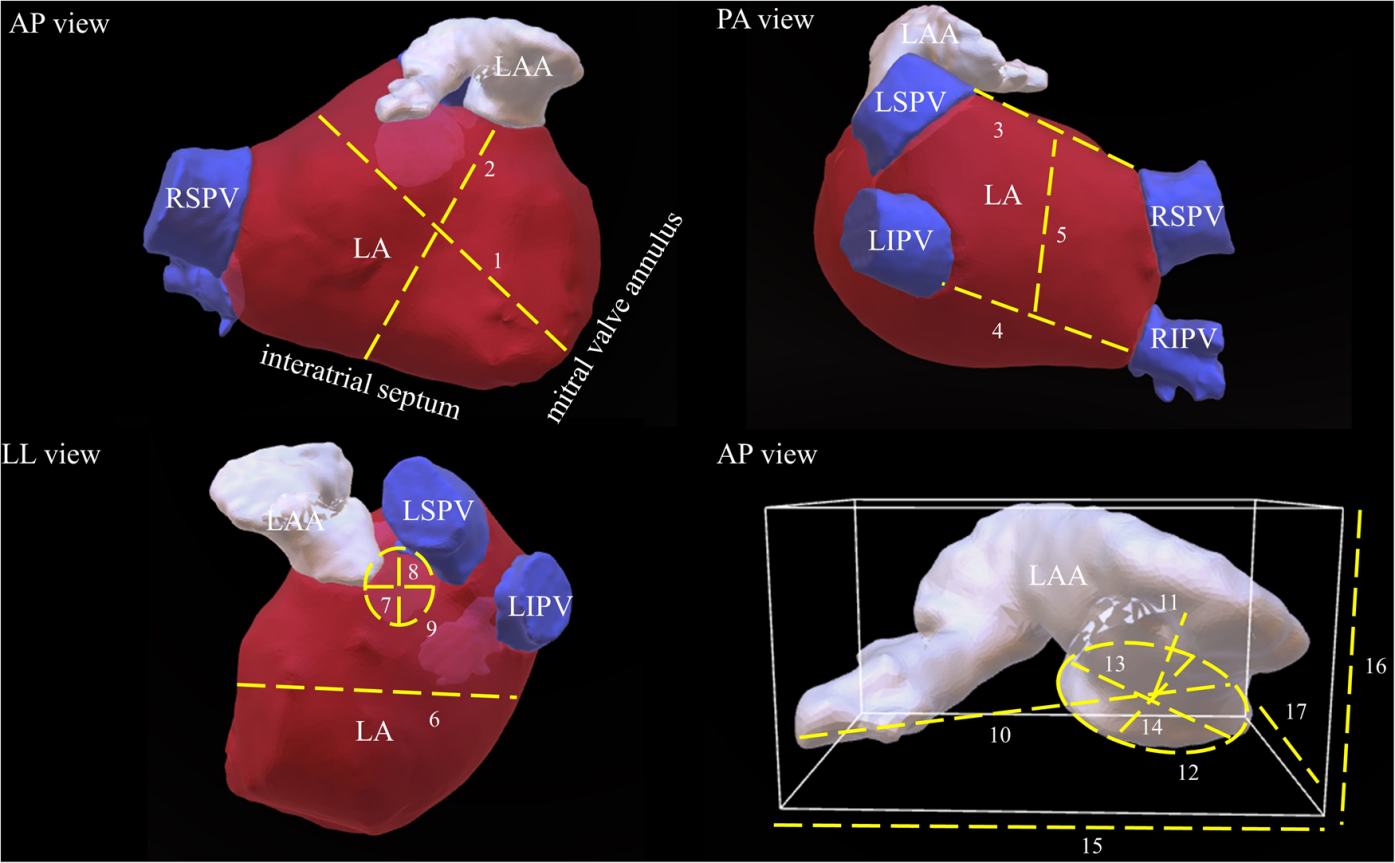
Schematic demonstration of important LA and LAA measurements. This picture illustrates most important LA and LAA dimensions in respective three-dimensional reconstructed CCTA images. The dotted lines and their respective numbers indicate the different measurements. 1: distance of the mitral valve annulus to the LA roof, 2: septum-orifice distance, 3: roof top line, 4: roof bottom line, 5: posterior wall box height, 6: LA depth, 7: max. ostial diameter of the RSPV, 8: minimal ostial diameter of the RSPV, 9: area of the RSPV, 10: LAA length, 11: distance to the first bend, 12: are of the LAA ostium, 13: maximal diameter of the LAA ostium, 14: minimal diameter of the LAA ostium, 15: maximal LAA width, 16: maximal LAA height, 17: maximal LAA depth. LAA, left atrial appendage; LA, left atrium; LSPV, left superior pulmonary vein; LIPV, left inferior pulmonary vein; RSPV, right superior pulmonary vein; RIPV, right inferior pulmonary vein.

**Figure 2:**
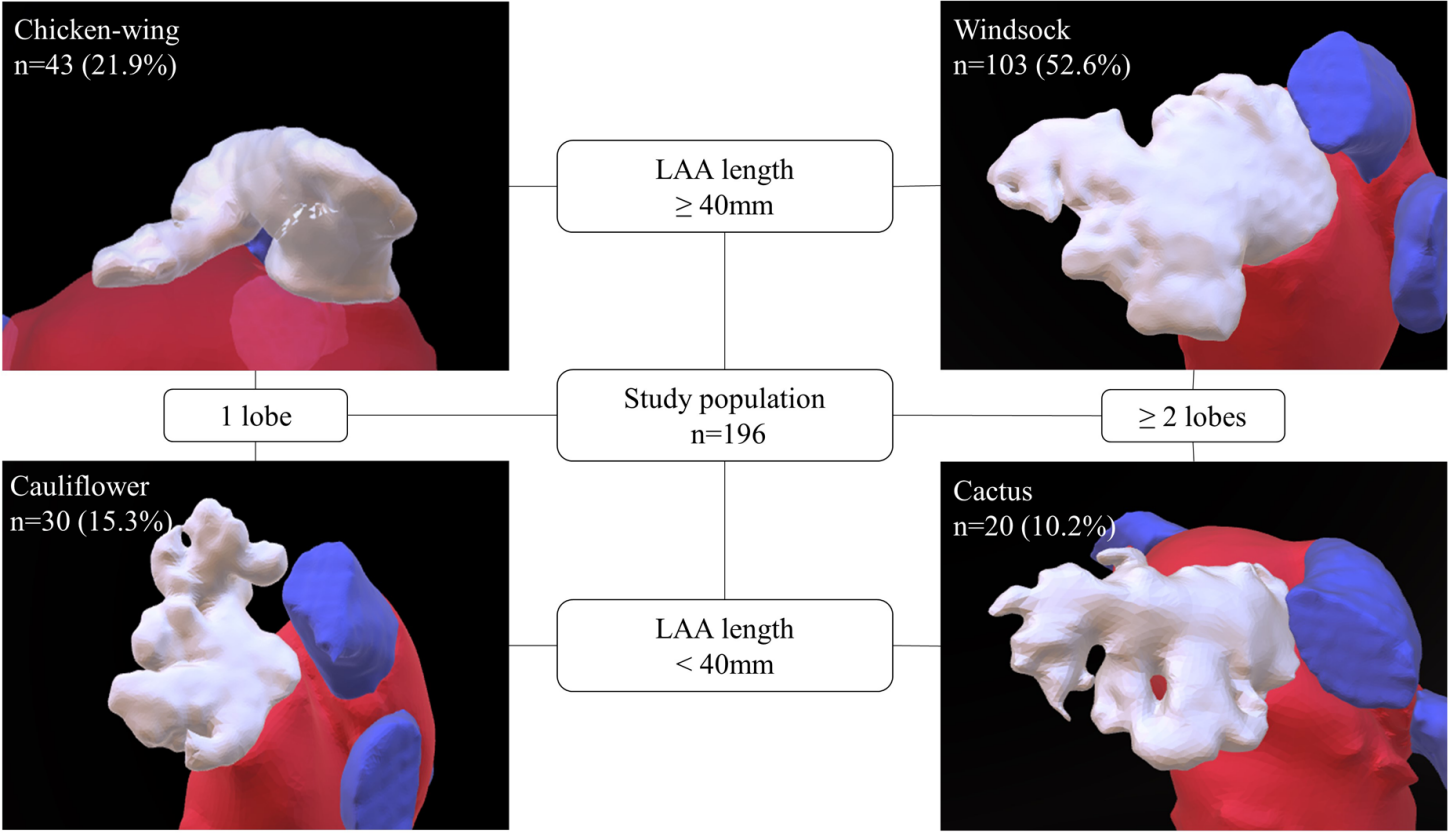
Classification of LAA morphology. This figure depicts the LAA morphological types as defined by Wang (20) with Kimura's quantitative qualifiers (21). According to the measured LAA length and number of lobes, LAA morphology was classified into one of four types: windsock, chicken-wing, cactus, and cauliflower. The prevalent LAA type distribution in the study is provided. LAA, left atrial appendage.

All baseline characteristics were acquired from the institutional registry entries and from the individual medical records. Measurement data acquisition deriving from CCTA images was previously described in detail (17). If there was poor LAA contrast, despite the absence of LAA thrombi, impairing LAA classification or LAA measuring, CCTA images were excluded. Data evaluated comprised two-dimensional (2D) and after multiplan volume-rendered post-processing also three-dimensional (3D) measurements. Major 2D parameters included were the maximal LAA height, length and depth. Most important parameters analyzed in 3D were the LA as well as the LAA volume, the LAA length, the roof top and roof bottom line, the septum-orifice distance and the distance of the mitral valve annulus to the middle of the LA roof (see Figure 1 for details). Morphological classification of the LAA was done according to the criteria established by Wang et al. and Kimura et al. into four different types: chicken-wing, windsock, cactus, and cauliflower (see Figure 2 for details) (20, 21).

### Study population

**Figure 3:**
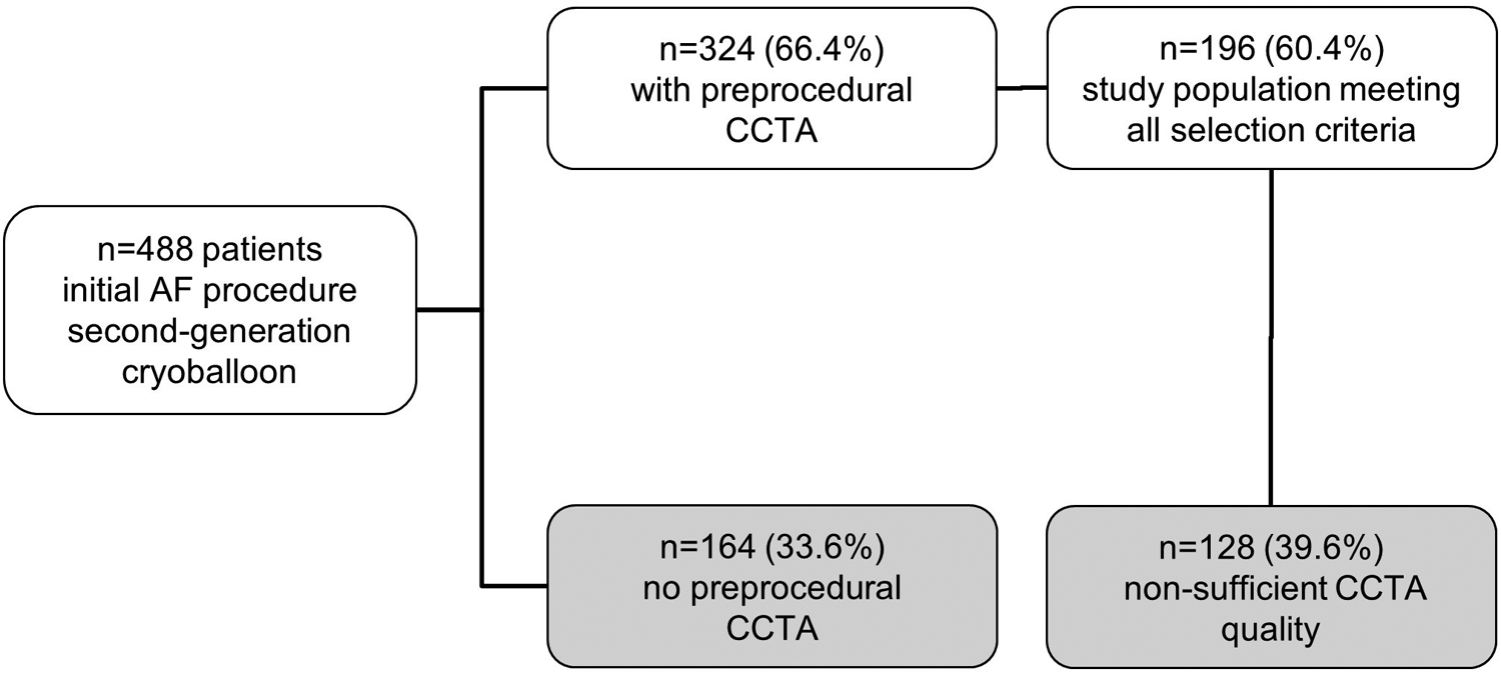
Study population—selection criteria. This flow chart explains the selection process of the study population. The top box shows the number of all patients included at the beginning. Each branching demonstrates one step of selection. CCTA, cardiac computed tomography angiography.

Between May 2012 and September 2016, 488 patients underwent CBA as the initial ablation procedure for symptomatic persAF. Pre-procedural CCTA was performed in 324 (66.4%) patients, and in 196 (60.4%) of patients CCTA images were of sufficient quality for measuring. All patients received complete electrical isolation of PV. Mean age was 65.7 ± 9.45 years and 69 (35%) patients were females. During a median follow-up of 19 (13; 29) months, 82/196 (41.8%) patients suffered from arrhythmia recurrence (excluding typical right atrial flutter or AVNRT). This group of patients were designated Group A. Patients free from any atrial arrhythmia recurrence following ablation were designated Group B (114/196 (58.2%)). A flow chart explaining the selection criteria is depicted in Figure 3.

### Impact of baseline characteristics and procedural results on outcome

The comparison of the baseline characteristics of the study population in terms of recurrence (Group A) and non-recurrence (Group B) are illustrated in Table 1. Baseline characteristics associated with atrial arrhythmia recurrence were secondary mitral regurgitation (MR) ≥grade 2 (*p* = 0.002), structural heart disease (*p* = 0.008) as well as hypertensive heart disease (*p* = 0.029). Other clinical factors such as the presence of diabetes mellitus (*p* = 0.16), impaired kidney function (*p* = 0.63), obstructive sleep apnea (*p* = 0.52) or AF history (*p* = 0.84) showed no statistical influence.

**Table 1:** Baseline characteristics .

	All patients	Group A (*n* = 82)	Group B (*n* = 114)	*p*-value
	*n* = 196	(with AA recurrence)	(without AA recurrence)	
Age, years	65.65 ± 9.46	66.5 ± 9.36	65.04 ± 9.5	0.31
Females (%)	69 (35)	32 (16.3)	37 (18.7)	0.23
Mitral regurgitation ≥°II (%)^c^	13 (6.6)	9 (4.6)	4 (2)	0.002
LA a-*p* diameter, mm^a^	45 [41; 50]	46 [43.5; 52]	44 [40; 48]	0.008
Ejection fraction (%)	55 [55; 60]	55 [55; 60]	55 [55; 60]	0.76
Diabetes mellitus (%)	11 (5.6)	6 (3.1)	5 (2.5)	0.16
GFR, ml/min	70 [60; 82]	67 [60; 83]	71 [60; 82]	0.99
Creatinine, mg/dl	0.9 [0.9; 1.1]	0.9 [0.9; 1.1]	0.9 [0.8; 1.1]	0.63
CHA_2_DS_2_-VASc-Score	3 [2; 4]	3 [2; 4]	3 [2; 4]	0.25
OSA (%)	9 (4.6)	6 (3.1)	3 (1.5)	0.52
Hypertension (%)	130 (66.3)	58 (29.6)	72 (36.7)	0.16
Structural heart disease (%)	82 (41.8)	41 (20.9)	41 (20.9)	0.008
Hypertensive heart disease (%)	51 (26)	27 (13.7)	24 (12.2)	0.029
Cardiomyopathy (%)	9 (4.6)	6 (3)	3 (1.5)	0.78
Overweight (BMI > 25) (%)	101 (61.5)	42 (25.6)	59 (35.9)	0.32
Obese (BMI > 30) (%)	30 (18.3)	13 (7.9)	17 (10.4)	0.67
Obese (BMI > 35) (%)	8 (4.9)	4 (2.4)	4 (2.4)	0.97
Left common ostium^b^ (%)	44 (22.4)	20 (10.1)	24 (12.2)	0.90
Accessory veins^b^ (%)	35 (17.9)	17 (8.7)	18 (9.1)	0.45
AF-History, months	37 [14; 68]	40 [20; 68]	30 [9; 70]	0.84

*n* (%), Mean ± SD, or Median (IQR).

AA, atrial arrhythmia; BMI, body mass index; LA, left atrium; GFR, glomerular filtration rate; OSAS, obstructive sleep apnea; a-p, anterior-posterior.

^a^ Determined by transthoracic echocardiography in parasternal view by 2D or M-Mode.

^b^ Determined by contrast enhanced cardiac computer tomography.

^c^ Classified as secondary mitral regurgitation.

Anatomical variances of the pulmonary veins, such as common ostiae (*p* = 0.90) or accessory pulmonary veins (*p* = 0.45) did also not have any impact on the risk of atrial arrhythmia recurrence.

There were no procedural data associated with atrial arrhythmia recurrence. See Supplement appendix for details.

### LAA morphology and arrhythmia recurrence

Comparing LAA morphologies, chicken-wing morphology had the largest overall volumes with a median LAA volume of 10.4 (8.6; 14.2) ml and an LA volume of 132.2 (114.1; 153.6) ml. Second largest LAA type was the windsock type with an LAA volume of 9.9 (8.1; 14.1) ml and an LA volume of 129.4 (109.9; 149.1) ml. Cauliflower and cactus types were significantly (*p* < 0.001) smaller, with LAA volumes of 6 (4.68; 8.3) ml and 6.7 (5.5; 7.7) ml and LA volumes of 111.8 (95.9; 136.8) ml and 103 (88.9; 126.7) ml. Figure 2 illustrates the distribution of the different LAA morphological types in the study population.

**Figure 4:**
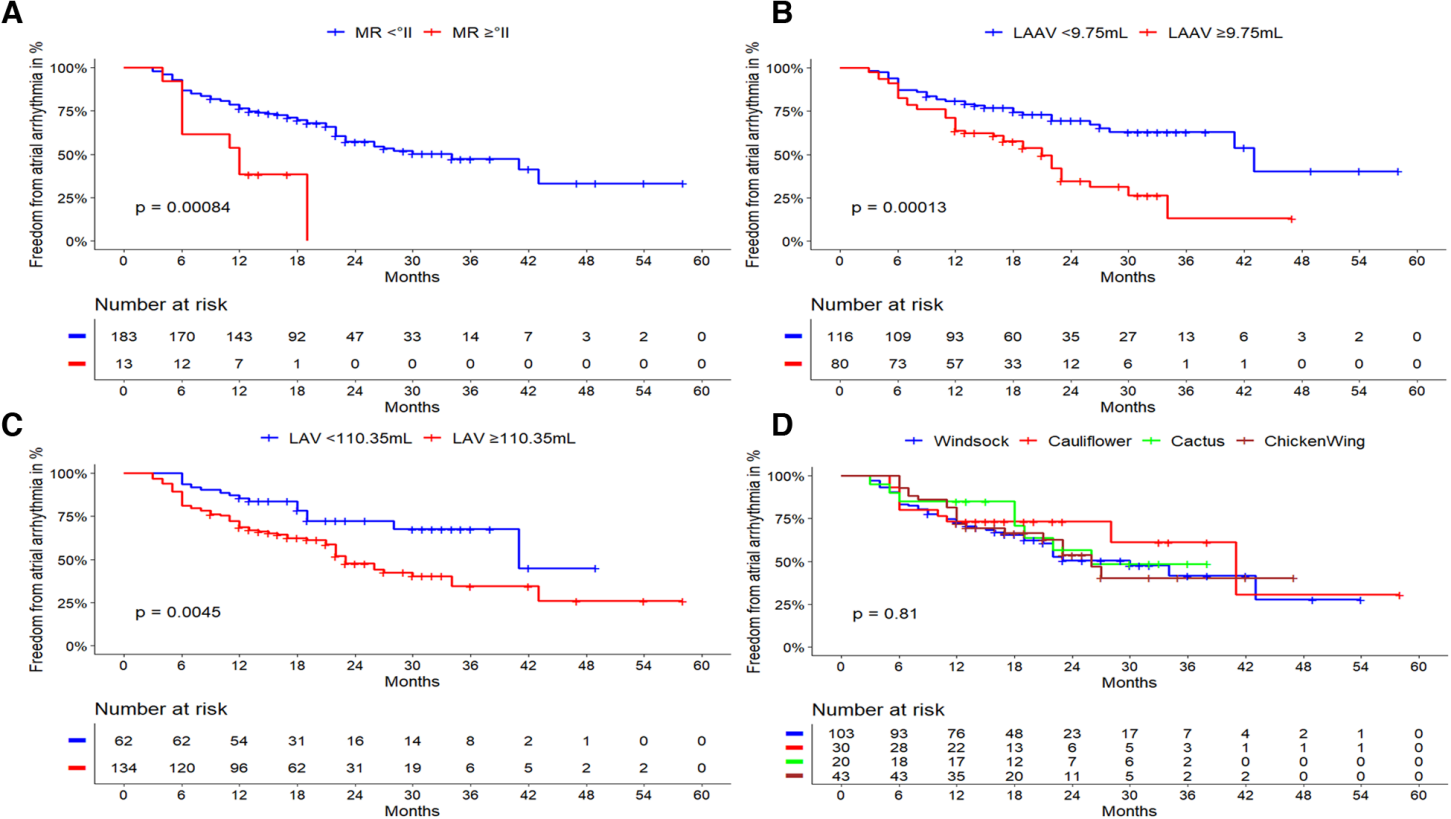
Outcome of cryoballoon ablation related to clinical predictors and LA/LAA anatomy. Kaplan-Meier curves regarding freedom from atrial arrhythmia after cryoballoon ablation according to (**A**): LAA volume cut-off level of ≥9.75 ml: Larger LAA volumes demonstrated a highly significant impact on the atrial arrhythmia recurrence rate after cryoballoon ablation (*p* < 0.001). (**B**): LA volume with a cut-off level of ≥110.35 ml: Larger LA volumes demonstrated a significant impact on the atrial arrhythmia recurrence rate after cryoballoon ablation (*p* = 0.0045). (**C**): mitral regurgitation ≥grade 2. Its presence was significantly related to a higher atrial arrhythmia recurrence rate (log-rank; *p* = 0.001). (**D**): LAA morphology, there was no statistical impact on the atrial arrhythmia recurrence rate after cryoballoon ablation (log-rank; *p* = 0.814). LAA, left atrial appendage; LA, left atrium; AF, atrial fibrillation; AT, atrial tachycardia; LAAV, left atrial volume; LAV, left atrial volume; MR, mitral regurgitation.

LAA morphology was not predictive for arrhythmia recurrence (log-rank, *p* = 0.814). Patients with chicken-wing morphology had a recurrence rate of 44.2%, followed by patients with windsock (43.7%), cactus (40%), and cauliflower morphology (33.3%). The respective Kaplan Meier plot is depicted in Figure 4D.

### CCTA derived LA and LAA parameters as predictors of atrial arrhythmia recurrence

Significant continuous parameters predicting arrythmia recurrence in univariate regression analysis were LAA volume (*p* < 0.001), LA volume (*p* = 0.003), area of the LAA ostium (*p* = 0.004), the perimeter of the LAA ostium (*p* = 0.006), maximal diameter of the LAA ostium (*p* = 0.009) and septum orifice distance (*p* = 0.011). LA anterior-posterior diameter measured by TTE was predictive for atrial arrhythmia recurrence (*p* = 0.008). Further statistically significant parameters are illustrated in Table 2.

**Table 2:** CCTA results of LA and LAA anatomy.

	All patients (*n* = 196)	Group A (*n* = 82)	Group B (*n* = 114)	*p*-value
		(with AA recurrence)	(without AA recurrence)	
LAA morphology				
Chicken-wing, %	43 (21.9)	19 (9.7)	24 (12.2)	0**.**90
Windsock, %	103 (52.6)	58 (29.6)	45 (23.0)	0**.**59
Cactus, %	20 (10.2)	8 (4.0)	12 (6.1)	0**.**48
Cauliflower, %	30 (15.3)	10 (5.1)	20 (10.2)	0**.**42
Significant LAA measurements				
LAA max width, mm	37.23 [33.5; 41.6]	38.4 [35.1; 42.9]	36.57 [32.6; 41.2]	0**.**028
LAA maximal depth, mm	40.07 [34.9; 45.7]	41.4 [35.9; 48.1]	39.0 [33.7; 44.4]	0**.**014
LAA volume, mL	10 ± 4.3	11.2 ± 4.6	9.16 ± 3.8	<0**.**001
LAA Dmax 3D, mm	25 [23; 28]	26 [23; 31]	25 [22; 27]	0**.**009
LAA Dmin 3D, mm	18 [16; 21]	18 [16; 21]	18 [15.8; 20]	0**.**038
LAA length, mm	45 [40; 49]	46.5 [40; 52]	42.5 [39.8; 47.3]	0**.**014
Perimeter LAA ostium, mm	68.84 [62; 76.2]	70.38 [63.7; 80.6]	67.89 [61.7; 74.5]	0**.**006
Area LAA ostium, mm²	378.9 ± 139	405.0 ± 152.8	360.1 ± 125.7	0**.**004
Significant LA Measurements				
LA volume, mL	128.8 ± 35.6	137.4 ± 42.4	122.5 ± 28.5	0**.**003
Depth of the LA, mm	40 [36; 44]	41 [37.8; 45]	39 [35; 43]	0**.**039
Septum orifice distance, mm	58 [55; 62.8]	60 [56.8; 64]	57.5 [54.75; 62]	0**.**011
Trapezoid area of the posterior LA wall, cm²	12.76 [11.0; 15]	13.06 [11.5; 15.7]	12.23 [10.5; 14.9]	0**.**033

*n* (%), Mean ± SD, or Median [IQR].

AA, atrial arrhythmia; LAA, left atrial appendage; LA, left atrium; MVA, mitral valve annulus; LIPV, left inferior pulmonary vein.

There was a strong correlation between LA volume, LAA volume and their respective companion parameters: LAA- and pulmonary vein ostial dimensions, septum orifice distance, roof bottom and roof top lines as well as distance from the mitral valve annulus to the middle of the LA roof. Among baseline parameters, kidney function, sex and age were significantly correlated with LA volume. LAA volume showed different significant associations i.e., with AF history, structural heart disease and MR ≥grade 2. Regarding LAA morphology, only cactus and chicken wing types were statistically correlated with LA volume, but all LAA morphologies correlated with LAA volume. A linear regression model was used to quantitatively demonstrate the relationship between LA and LAA volume. The results show, that LAA volume depends on LA volume and increases by 0.60 ml per 10 ml increase of LA volume (*p* < 0.001, see Supplementary Appendix Figure S1).

### Independent predictors for atrial arrhythmia recurrence

Taking all previously identified risk factors and intervariable relationships into consideration, multivariate cox regression analysis revealed two main statistically independent risk factors for atrial arrhythmia recurrence as shown in Table 3. The most significant parameters were the LAA volume [HR 1.082; 95% CI (1.032–1.134); *p* = 0.001], followed by the presence of MR ≥grade 2 [HR 2.495; 95% CI (1.207–5.126); *p* = 0.013]. Per one milliliter increase of the LAA volume, the risk of recurrence increases by 8.2%. Both independent risk factors were also evaluated using Kaplan Meier survival curves for atrial arrhythmia recurrence during follow-up (Figure 4).

**Table 3:** Evaluation of independent risk factors.

	Univariate Analysis			Multivariate Analysis		
	HR	95% CI	*p*-value	HR	95% CI	*p*-value
Baseline Characteristics						
Female sex	1.317	0.842–2.060	0.227			
Age, years	1.012	0.989–1.036	0.305			
Mitral regurgitation ≥°II	3.090	1.517–6.290	0.002	2.495	1.207–5.126	0.013
Structural heart disease	1.802	1.163–2.793	0.008			
Weight, kg	0.990	0.975–1.005	0.194			
Height, cm	0.089	0.007–1.189	0.068			
CCTA Measurement Data						
LAA volume, mL	1.089	1.041–1.140	<0.001	1.082	1.032–1.134	0.001

Univariate analysis of baseline characteristics and measurement data according freedom of AF/AT after cryoballoon ablation provided multiple highly significant parameters. To prevent problems of multicollinearity in multivariate models, CCTA measures were reduced to LAA volume as the most significant parameter. After stepwise multiple regression with bidirectional elimination two parameters could be identified as independent risk factors: LAA volume and mitral regurgitation ≥°II. The significance of the multivariate Cox regression model was *p* < 0.001. AF, atrial fibrillation; AT, atrial tachycardia; CCTA, cardiac computed tomography angiography; LAA, left atrial appendage.

### Cut-off values for LAA and LA volumes predicting recurrence after CBA

Using ROC analysis, cut-off values for LAA and LA volumes were determined. LAA volumes ≥9.75 ml [sensitivity 0.56, specificity 0.69, area under the curve (AUC) 0.64] and LA volumes ≥110.35 ml (sensitivity 0.81, and specificity 0.40, AUC 0.62) were associated with atrial arrhythmia recurrence. Subsequently, Kaplan Meier plots were calculated, in which both cut-off values for LAA volume (*p* < 0.001) and LA volume (*p* = 0.004) showed high statistical significance (Figures 4A,B).

## Discussion

To our knowledge, this study is the first to establish an independent association between the volume of the LAA and the recurrence of AF after CBA in patients with persAF. It highlights that the LAA volume is a more reliable predictor than the LA volume in persAF patients after CB-PVI. Therefore, the emphasis on LAA volume should be significantly increased in future clinical research involving these patients.

However, the individual shape of the LAA was not predictive. We have demonstrated that CBA is safe and effective as a treatment strategy in persAF patients with an overall low complication rate and a reasonable success rate.

Although, CCTA can provide useful additional information to predict the success rate of the ablation procedure and may also be used to develop individual treatment strategies beyond PVI in persAF patients experiencing atrial arrhythmia recurrence, we recommend TTE to be used as the primary pre-procedural imaging modality in clinical practice as it is widely available, without the need for radiation or contrast dye.

### LAA volume as the primary independent predictor of atrial arrhythmia recurrence

The LAA volume is, besides the presence of MR ≥grade 2, the primary independent risk factors for arrhythmia recurrence in persAF following successful cryoballoon PVI. Some previous trials already indicated that larger LAA volumes may be associated with arrhythmia recurrences after RF ablation in persAF patients (22). However, up to now, an enlarged LA volume was considered to be the more relevant predictor (23, 24). Rising wall stress due to increasing LA volumes can promote myocyte hypertrophy and interstitial fibrosis via the cardiac endothelin-1 receptors (25). These areas of slow conduction and altered repolarization dynamics can stabilize rotors, singularities around which spiral waves disorganize and fuse passively with the milieu. Ultimately, this may lead to functional reentries (26). But also, the LAA structure is influenced by AF as it has a highly complex architecture made by pectinate muscles lining the endocardial surface (27). Studies revealed that structural remodeling due to AF leads to a reduction in the number of pectinate muscles and can therefore promote the origin of reentries (28). Hocini et al. have already described that in 11% of patients undergoing ablation of persAF, atrial tachycardia originated in the LAA in the form of localized reentries (15). In contrary, a recent study showed only a very low incidence (0.3%) of true LAA triggers in patients undergoing catheter ablation (29).

The BELIEF trial indicated that PVI plus empirical LAA isolation could increase clinical success in patients with persAF (30). However, LAA isolation causes also electromechanical dissociation from the LA, and thus increases the risk for LAA thrombus formation and thromboembolism.

Our study suggests that the role of the LAA and in particular the LAA volume requires increasing attention in developing novel treatment options for persAF patients. The present data suggest that especially patients with persAF and large LAA should be studied in AF ablation trials focusing on LAA as a target for ablation in PVI non-responders. More precisely, patients with an LAA size ≥9.75 ml could represent candidates for additional ablation beyond standard PVI. Future studies need to investigate, whether persAF patients with large LAA would benefit from additional ablation beyond PVI e.g., LAA isolation with subsequent LAA closure.

### Other clinical predictors of atrial arrhythmia recurrence

Besides LA volume, the second independent predictor of atrial arrhythmia recurrence after CBA was identified to be the presence of MR ≥grade 2. This finding is in line with Gertz et al. (31). It is widely known that the presence of MR leads to increased LA volumes due to volume overload and thus ultimately promoting atrial remodeling (32). Through a change in electrophysiologic properties MR is therefore associated not only with the origination but also the maintenance of AF (33–35).

Certain clinical characteristics, such as diabetes mellitus, impaired kidney function as well as obstructive sleep apnea, were not linked to atrial arrhythmia recurrence as previously stated (36–38). Reason for this might be the relatively small number of patients in these categories. In accordance to Khoueiry et al. (39), PV size as well as anatomical variations such as common ostiae and additional pulmonary veins were also not associated with atrial arrhythmia recurrence in the present study. In addition, we did not find procedural predictors for recurrences. These findings underline the overall efficacy of cryoballoon PVI even in challenging anatomies.

### Impact of LAA morphology

Di Biase et al. stated that LAA morphology has significant impact on the risk of stroke in patients with AF with chicken wing morphology having the lowest chance for thromboembolic events (40). However, the influence of LAA morphology on the risk of recurrence after an ablation procedure is uncertain. A recently published trial by Kogicit et al. suggested that the cauliflower type may be associated with a two-fold increased risk of recurrence after CBA (41). Our study could not demonstrate any statistical association between any type of LAA morphology and atrial arrhythmia recurrence after CBA. Notably, our study population only included patients with persAF, which was not the case for Kogicit et al.

### Pre-procedural CCTA and ablation strategies beyond PVI

To ensure an optimal anatomic understanding of patients undergoing PVI, CCTA can be used upfront for the identification and detection of PV variants and other LA features. Usually, intraprocedural LA angiography, three-dimensional electroanatomic mapping of the LA, or intracardiac echocardiography is used to determine LA and PV anatomy. Beside the precise evaluation of PV anatomy, CCTA can help exclude CAD as well as rule out possible thrombus formation prior to the procedure (42). Up to now it is unclear whether pre-procedural CCTA can improve the outcome of initial cryoballoon ablation. CCTA has two main limitations: radiation exposure for otherwise healthy patients as well as contrast media containing iodine that might impair kidney function or thyroid hormone balance. Therefore, TTE and TEE is usually the primary pre-procedural imaging modality due to its low cost, high availability and sufficient measurements without the use of radiation or contrast agent. Especially when considering non-volumetric LA measurements, parameters obtained through echocardiography, such as the anterior-posterior LA diameter, exhibit similar utility and impact as non-volumetric CCTA parameters, such as the septum-orifice distance. The significance level regarding atrial arrhythmia recurrence appears to be equally strong for both modalities. Regarding precise LAA analysis however, echocardiography offers only limited options.

The STAR-AF II Trial has demonstrated that a PVI only strategy is equally effective as compared to PVI plus additional ablation as empirical lines or CFAE. The recently published DECAAF II trial and the ALICIA trial indicated that PVI plus pre-procedural magnetic resonance imaging guided fibrosis ablation showed no additional benefit as compared to a PVI only strategy in persAF patients (9, 43), however more cerebrovascular events were observed in the group with fibrosis ablation in addition to PVI.

Given the high significance of LAA volume revealed by our study, CCTA should be considered as an imaging modality for patients with persAF suffering from atrial arrhythmia recurrence following PVI in studies evaluating ablation strategies beyond PVI such as substrate modification or even LAA isolation.

### Limitations

There are some important limitations that need to be mentioned. This is a prospective single-center study with a retrospective evaluation and thus with inherent bias to this design. Prior to the AF ablation procedure not every patient underwent CCTA, thus the data is not fully consecutive. Moreover, image quality for precise LAA analysis was fluctuating, so in some patients, data assessment was more difficult than in others. CCTA measurements were typically performed after a period of preparatory fasting to ensure the highest possible image quality. Nevertheless, it is important to note that variations in volume loading could potentially affect the accuracy of measurements of the LA and LAA volume. The follow-up was based on the evaluation of symptoms, clinical visits, ECG and Holter monitoring (1–7days). There was no continuous monitoring with an implantable device available. As a result, asymptomatic atrial arrhythmia recurrences might be underestimated. There was no data assessment of possible LAA triggers during the AF ablation procedure, so we could show no evidence, whether the LAA is arrhythmogenic. Though LAA morphology classification was determined and validated by four different experts, the classification criteria in general are still rather subjective and imprecise.

## Conclusion

For symptomatic persAF patients, CB-PVI as the initial ablation procedure is safe and effective with reasonable outcomes. Main independent predictors for atrial arrhythmia recurrence were LAA volume and MR ≥grade 2. LA volume seemed to be an inferior risk factor in persAF patients following CBA as compared to LAA volume. It is of special interest that patients with LAA volumes ≥9.75 ml showed a strong association for arrhythmia recurrence. LAA morphology could not predict the outcome of CBA. TTE should be used as the primary pre-procedural imaging modality. Detailed CCTA analysis could provide useful additional information to predict the success rate of the ablation procedure and should be considered in further studies evaluating ablation strategies in PVI non-responders.

## Data Availability

The datasets mentioned in this article are accessible since they were stored on the server of the Heart-Center Munich-Bogenhausen, Munich, Germany. The authors assure that the data supporting the results of this study can be found within the article itself and /or its supplementary materials. Given the nature of this research, the participants did not provide consent for their data to be publicly shared, hence the availability of raw data is not possible. To request access to the datasets, please contact florian.straube@muenchen-klinik.de.
