# Supplementary material for: Left atrial appendage volume is an independent predictor of atrial arrhythmia recurrence following cryoballoon pulmonary vein isolation in persistent atrial fibrillation
Source: Front Cardiovasc Med. 2023 Jun 19;10:1190860. doi: 10.3389/fcvm.2023.1190860 (PMC10315839; doi:10.3389/fcvm.2023.1190860)
Supplement: Supplementary file 1 [file Datasheet1.docx]

**Supplement appendix**

**Results**

**Procedural results**

With a median procedural time of 130 (120; 160) min and a median LA time of 95 (80; 120) min. There were no additional RF or cryo-tip ablations required. The median dose area product was 1919 (1103; 3255) cGycm2, and the fluoroscopy time was 22 (17; 28) min. A total of 23 (11.7%) complications were documented including 14 (7.1%) peri-procedural complications and 9 (4.6%) post-procedural complications. Peri-procedural complications consisted of 14 (100%) transient phrenic nerve palsies. Post-procedural complications comprised 3 (1.5%) pericardial effusions, 2 (1%) puncture site hematomas, 1 (0.5%) fistula, 1 (0.5%) bronchitis, 1 (0.5%) respiratory tract infection, 1 (0.5%) gross haematuria and 1 (0.5%) puncture site oozing. No major adverse cardiovascular or cerebral event (MACCE) was observed. Pericardial effusions were treated conservatively. For further details see supplement table 1.

**Tables**

**Supplementary Table 1: Procedural results**

|  | **All patients (n=196)** | **Group A (n=82) (with AA recurrence)** | **Group B (n=114) (without AA recurrence)** | **p-value** |
| --- | --- | --- | --- | --- |
| **Procedural time, min** | 130 [120, 160] | 130 [120; 150] | 135 [118; 165] | 0.90 |
| **LA time, min** | 95 [80; 120] | 95 [85; 108] | 100 [80; 120] | 0.77 |
| **Fluoroscopy time, min** | 22 [17; 28] | 22 [16; 27] | 22 [18; 29] | 0.43 |
| **Dose area product, cGycm^2^** | 1919 [1103; 3255] | 2086 [1053; 3342] | 1885 [1140; 3171] | 0.19 |
| **Complications** |  | | | |
| **Postprocedural complication rate** | 9 (4.5) | 3 (3.7) | 6 (5.3) | 0.76 |
| **Pericardial effusion** | 3 (1.5) | 1 (2.1) | 2 (1.8) | - |
| **Puncture site hematoma** | 2 (1.0) | 1 (2.1) | 1 (0.9) | - |
| **Fistula** | 1 (0.5) | 0 (0.0) | 1 (0.9) | - |
| **Gross haematuria** | 1 (0.5) | 0 (0.0) | 1 (0.9) | - |
| **Respiratory tract infection** | 1 (0.5) | 1 (2.1) | 0 (0.0) | - |
| **Puncture site oozing** | 1 (0.5) | 0 (0.0) | 1 (0.9) | - |
| **Periprocedural complication rate** | 14 (7.1) | 3 (3.7) | 11 (9.7) | 0.16 |
| **Transient phrenic nerve palsy** | 14 (7.1) | 3 (3.7) | 11 (9.7) | 0.16 |

n (%), Mean ± SD, or Median [IQR]

AA: atrial arrhythmia, LA: left atrium, LA: left atrium

**Figures**

**Supplementary Figure 1: Correlation of LA and LAA volumes**

The figure shows a linear regression model of LAA volume and LA volume. It demonstrates that per 10 mL increase of LA volume, LAA volume increases by 0.6 mL. The significance level of the model was p<0.001.

LAA: left atrial appendage; LA: left atrium
