# Supplementary figures and images for: Left atrial appendage volume is an independent predictor of atrial arrhythmia recurrence following cryoballoon pulmonary vein isolation in persistent atrial fibrillation
Source: Front Cardiovasc Med. 2023 Jun 19;10:1190860. doi: 10.3389/fcvm.2023.1190860 (PMC10315839; doi:10.3389/fcvm.2023.1190860)

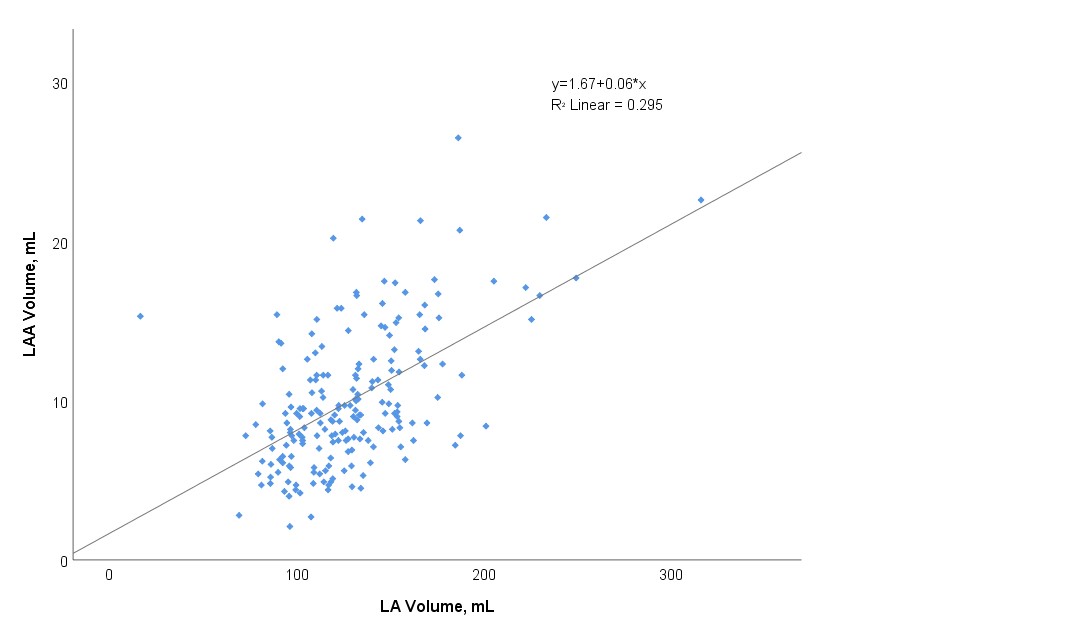

Supplement: Supplementary file 2 [file Image1.jpeg]
